# Relationship between polycomb‐group protein BMI‐1 and phosphatases regulating AKT phosphorylation level in endometrial cancer

**DOI:** 10.1111/jcmm.14782

**Published:** 2019-12-21

**Authors:** Agnieszka Zaczek, Paweł Jóźwiak, Piotr Ciesielski, Ewa Forma, Katarzyna Wójcik‐Krowiranda, Łukasz Cwonda, Andrzej Bieńkiewicz, Magdalena Bryś, Anna Krześlak

**Affiliations:** ^1^ Department of Cytobiochemistry Faculty of Biology and Environmental Protection University of Lodz Lodz Poland; ^2^ Clinical Division of Gynecological Oncology Medical University of Lodz Lodz Poland

**Keywords:** AKT, BMI‐1, endometrial cancer, PHLPP, PTEN

## Abstract

The PI3K/AKT pathway is frequently activated in endometrial carcinoma. BMI‐1 (B‐lymphoma Mo‐MLV insertion region 1) protein affects expression of PTEN (phosphatase and tensin homolog) in some cancers, but its significance for endometrial tumorigenesis is not known. The objective of this study was to determine the relationship between BMI‐1 and expression of factors affecting AKT (protein kinase B) phosphorylation level in endometrial cancer. The expression of proteins and mRNAs was investigated in endometrial cancer specimens and samples of non‐neoplastic endometrial tissue by Western blot and RT‐PCR, respectively. The impact of BMI‐1 down‐regulation on AKT phosphorylation and expression of genes coding for several phosphatases were studied in HEC1A cells. The results showed that BMI‐1 depletion caused increase in PHLPP1 and PHLPP2 (PH domain and leucine‐rich repeat protein phosphatases 1/2) expression and decrease in phospho‐AKT (pAKT) level. In more advanced tumours with higher metastatic potential, the expression of BMI‐1 was lower compared to tumours less advanced and without lymph node metastasis. There were significant inverse correlations between BMI‐1 and PHLPPs, especially PHLPP1 in normal endometrial samples. The inverse correlation between BMI‐1 and PHLPP1/PHLPP2 expression was observed in PTEN positive but not PTEN negative cancers. Low PHLPP2 expression in tumours predicted poorer overall survival. BMI‐1 impacts on AKT phosphorylation level in endometrial cells by regulation of PHLPP expression.

## 1 BACKGROUND

B‐lymphoma Mo‐MLV insertion region 1 (BMI‐1) is a component of polycomb repressive complex 1, which is involved in the regulation of genes activity. Several studies have shown that BMI‐1 expression is frequently altered in various types of human cancers, including gastric, ovarian, breast, head and neck, pancreatic and lung cancers.^1^, ^2^ It has been found that this protein plays an important role in cancer initiation and progression by promotion of cell growth and proliferation, inhibition of apoptosis as well as regulation of invasion and metastasis.^1^, ^2^ BMI‐1 is related to poor prognosis of non‐small cell lung cancer, ovarian cancer, head and neck cancer and haematological malignancies.^2^ It is suggested that one of the possible mechanisms by which BMI‐1 promotes tumorigenesis is the activation of PI3K/AKT pathway which is due to inhibition of PTEN expression.^3^, ^4^, ^5^ It has been shown that in nasopharyngeal cancer cells, PTEN inhibition and activation of the PI3K/AKT/GSK3β pathway by BMI‐1 causes stabilization of the transcription factor Snail1, which together with PRC1 binds to E‐cadherin promoter and down‐regulates its expression. It leads to reduction in the ability of cells to adhere and in consequence enhances their invasive potential.^3^ BMI‐1 promotes invasion and metastasis of pancreatic cancer stem cells by activating PI3K/AKT signalling.^5^ BMI‐1 is probably also involved in endometrial carcinogenesis, but data are limited and its exact function is unknown. Honig et al^6^ found that endometrial carcinomas express higher level of BMI‐1 than benign controls. Moreover, it has been found that miR‐194 and miR‐200 by targeting BMI‐1 inhibit epithelial to mesenchymal transition (EMT) of endometrial cancer cells.^7^, ^8^ In contrast to results suggesting function of BMI‐1 in EMT and metastasis, Engelsen et al^9^ demonstrated that low, rather than high BMI‐1 expression is associated with an aggressive phenotype in endometrial carcinomas.

Alterations of PI3K/AKT signalling pathway play essential role in endometrial carcinogenesis and currently a number of clinical trials are underway to assess the effectiveness of therapies using PI3K/Akt pathway inhibitors.^10^ The PI3K/AKT pathway is frequently activated in endometrial carcinoma, often due to PTEN loss. However, the significance of PTEN loss for endometrial cancer progression is not clear. Karnezis et al^11^ in study of large cohort of endometrial cancer found that association of PTEN loss with clinicopathological variables was poor and prognostic value of PTEN status very limited. PTEN indirectly impacts on AKT phosphorylation since it dephosphorylates the second messenger inositol 1,4,5‐triphosphate. Several other phosphatases negatively regulate the PI3/AKT pathway. Two isoforms of PH domain leucine‐rich repeat protein phosphatase (PHLPP), PHLPP1 and PHLPP2 have been shown to directly dephosphorylate AKT.^12^ There are no data concerning expression of PHLPPs in endometrial cancer. Thus, the further studies concerning regulation of PI3K/AKT pathway in endometrial cancer are needed.

The aim of this study was to estimate the BMI‐1 expression in normal and cancerous endometrial tissues and determine if there is any association between BMI‐1, PTEN and phosphorylation level of AKT. Moreover, we analysed the impact of BMI‐1 silencing on expression of several phosphatases involved in direct or indirect activation of AKT. The correlation of BMI‐1 and PHLPPs in endometrial normal and cancer tissues was analysed.

## 2 MATERIAL AND METHODS

### 2.1 Patients and tissue samples

The study included 37 normal endometrial tissue samples and 93 endometrial cancer samples, which were taken from patients of Department of Gynecological Oncology Copernicus Memorial Hospital, Lodz, Poland. Samples of normal endometrial tissues were obtained from patients, who had undergone hysterectomy due to leiomyoma or a uterus prolapse. The tumour stage and histologic diagnosis of each patient were classified under International Federation of Gynecology and Obstetrics (FIGO) 2009 criteria^13^ and the World Health Organization histologic type classification system, respectively. The tumours were graded as well‐differentiated (G1), moderately‐differentiated (G2) or poorly differentiated (G3). Clinicopathological characteristics of endometrial cancer samples are presented in Table 1. The study proposal was approved by the Ethics Committee of University of Lodz (18/KBN‐UŁ/ I/ 2017).

**Table 1:** Characteristics of patients and endometrial cancers

Characteristics	Number of patients (%)
Control samples	37
Patients age (49.96 ± 12.99)	
Endometrial cancer samples	93
Patients age (63.43 ± 10.48)	
FIGO stage	
I	51 (54.8)
II	19 (20.4)
III	21 (22.6)
IV	2 (2.2)
Histological grade	
G1	17 (18.3)
G2	52 (55.9)
G3	24 (25.8)
Lymph node metastasis	
No	77 (82.8)
Yes	16 (17.2)

### 2.2 Cell culture and treatment

HEC1A cells were obtained from the American Type Culture Collection. Cells were cultured in DMEM:F12 media (Life Technologies) containing 10% (v/v) FBS at 37°C and 5% CO_2_. Knock‐down experiments were performed using Silencer Select siRNA (ID: S2016) (Ambion^®^). Cells were seeded in a 6‐well tissue culture plates at a density of 6 × 10^5^ cells/well. To knockdown of BMI‐1, for each well 30 nmol/L control siRNA or siRNA targeting BMI‐1 was complexed to lipofectamine RNAiMAX (Invitrogen^™^; Thermo Fisher Scientific) following manufacturer's specifications. The effect of BMI‐1 silencing was check after 48 hours.

### 2.3 RNA extraction, cDNA synthesis and real‐time quantitative PCR

Total RNA from endometrial samples or cells was isolated using Trizol^®^ Reagent (Sigma Aldrich) following manufacturer's protocol and quantified spectrophotometrically. The reverse transcription reaction was performed using the High Capacity cDNA Reverse Transcription Kit (Applied Biosystem) according to manufacturer's protocol. Amplification of the cDNA was performed using TaqMan^®^ Gene Expression Assay (Thermo Fisher Scientific) according to the manufacturer's instructions.

The fluorogenic, FAM‐labelled probes and the sequence‐specific primers for *BMI1*, *PTEN*, *PHLPP1*, *PHLPP2*, *PP2A*,*INPP4B*, *INPP5D* and the internal control *HPRT1* were obtained as inventoried assays: Hs00180411_m1, Hs01597871_m1, Hs00982295_m1, Hs00603515_m1, Hs01038089_m1, Hs00183290_m1 and Hs02800695_m1 (Applied Biosystems; ThermoFisher Scientific). Fold differences in genes expression, normalized to HPRT1 levels were calculated using the formula 2^ΔΔCt^.

Analysis of PTEN and PHLPP1/2 expression in endometrial tissues was performed using HS RT‐PCR Master Mix SYBR B (A & A Biotechnology, Poland) according to the manufacturer's protocol. The *HPRT1* gene was used as internal control. The PCR primer sequences were designed according to the human *PTEN, PHLPP1, PHLPP2* and HPRT1 gene sequences reported in GeneBank and were synthesized as follows: PTEN_F: ACAGCCATCATCAAAGAGATCGT, PTEN_R: TGCTTTGAATCCAAAAACCTTACTA

HPRT1_F: CCCTGGCGTCGTGATTAGTG, HPRT1_R: ACACCCTTTCCAAATCCTCAGC, PHLPP1_F: AAACCTCACAGCACGGGTAG,

PHLPP1_R: AGGCAGGTCCCACATAGGAT, PHLPP2_F: TCCTGACCTCGGCTGTATGA, PHLPP2_R: GGGTCTTTCCCTTGCGTACA. The RT‐qPCR reaction was performed using Mastercycler^®^ep realplex (Eppendorf). The relative expression levels of gene were calculated using ΔCt method. ΔCt (Ct_gene_—Ct_HPRT_) values were recalculated into relative copy number values (number of gene mRNA copies per 1000 copies of *HPRT1*mRNA). Relative gene expression in cells was calculated using ΔΔCT normalized to*HPRT1* gene expression.

### 2.4 Western blot and densitometric analysis

Endometrial tissue samples were lysed in a RIPA buffer (50 mmol/L Tris HCl pH 8, 150 mmol/L NaCl, 1% Nonidet P‐40, 0.5% sodium deoxycholate, 0.1% SDS, 1 mmol/L EDTA, 1 mmol/L PMSF). Protein concentration was determined using Lowry method. Protein lysates (60 µg) were resolved by 8% SDS‐PAGE and transferred to immobilon P membranes. The membranes were incubated for 2 hours at room temperature with following primary antibodies: anti‐BMI‐1 (diluted 1:1000; Cell Signaling Technology), anti‐Phospho‐Akt (Ser473) (diluted 1:1000; Cell Signaling Technology), anti‐PTEN and anti‐AKT (diluted 1:1000; Santa Cruz Biotechnology). After washing with TBST (Tris‐buffered saline with Tween‐20), immunoblots were incubated 1 hour at room temperature with appropriate secondary antibodies conjugated with horseradish peroxidase (diluted 1:5000; Cell Signaling Technology). The blots after stripping were reprobed with anti‐β‐actin antibodies (1:1000; Santa Cruz Biotechnology). The bands corresponding to proteins were analysed in Gel Pro 3.0 Analyzer software (Media Cybernetics) by measuring integrated optical density (IOD) of the bands. To avoid the errors due to transfer and handling differences, we applied in lane normalization using β‐actin as an internal reference. Moreover, the same reference sample was applied to each blot (external control). This normal endometrial sample was chosen in the first Western blotting experiments since it showed median expression of studied proteins. The results are presented as a relative IOD, that is the ratio of specific protein IOD to β‐actin IOD and reference sample. This procedure allowed us to compare the bands on different blots and avoid the errors due to possible differences in ECL signal. The AKT phosphorylation level is expressed as phospho‐AKT/AKT ratio.

### 2.5 Statistical analysis

Statistical analysis of the results was performed using the statistical program GraphPad Prism 5.0 (GraphPad Software Inc). The non‐parametric Mann‐Whitney *U* test was used when two groups were compared. Comparisons between more than two groups were done using Kruskal‐Wallis test. For pairwise multiple comparisons, Dunn's post hoc test was used. Spearman correlation coefficient was calculated for correlation analysis. The Student's paired ‐test was used to compare the differences between treated and untreated cells. A *P*‐value <.05 was considered to indicate a statistically significant difference.

## 3 RESULTS

### 3.1 Down‐regulation of BMI‐1 in HEC1A cells

**Figure 1:**
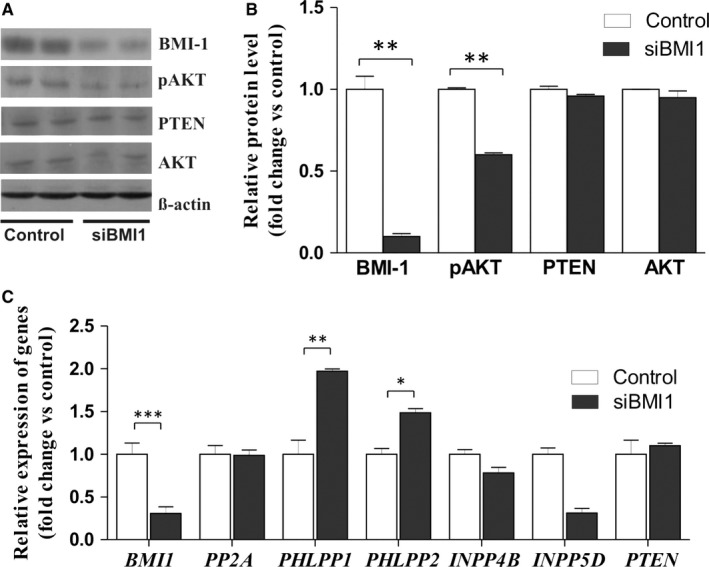
Effect of BMI‐1 down‐regulation on AKT phosphorylation level and expression of phosphatases in HEC1A cells. A, Representative immunoblots showing BMI‐1, PTEN, AKT proteins and phosphorylated AKT in HEC1A cells treated for 48 h with 30 nmol/L BMI‐1 siRNA or scrambled siRNA (control). B, Bar graph shows the densitometric analysis of BMI‐1, PTEN, AKT and phosphorylated AKT (pAKT) levels in cells treated with BMI‐1 siRNA and scrambled siRNA and represents the mean ± SD of three independent experiments. C, Relative changes in *BMI1, PP2A, PHLPP1, PHLPP2, INPP4B, INPP5D* and *PTEN* mRNAs expression levels in siRNA treated cells compared to untreated cells; bar graph represents the mean ± SD. ^*^ *P* < .05, ^**^ *P* < .01

To assess the impact of BMI‐1 on PTEN and AKT, the expression the *BMI1* was depleted in endometrial cancer cell line HEC1A by siRNA. In cells treated with siRNA, the BMI‐1 transcript level was substantially reduced (by 70%‐80%). The protein level of BMI‐1 was also very low (Figure 1A,B). In cells with BMI‐1 depletion, a marked change in AKT phosphorylation level (pAKT) was found. However, the decrease in pAKT was not associated with PTEN since the cells with BMI‐1 down‐regulation did not show any change in PTEN expression level (Figure1B). Thus, we analysed the impact of BMI‐1 depletion on mRNA expression of several phosphatases involved in direct and indirect AKT regulation, that is *PTEN, PP2A *(protein phosphatase 2A)*, PHLPP1, PHLPP2, INPP4B *(inositol polyphosphate‐4‐phosphatase type II B) and *INPP5D* (inositol polyphosphate‐5‐phosphatase D). The results showed a significant increase in PHLPP mRNA expression, especially PHLPP1. The down‐regulation of BMI‐1 caused 2‐fold increase of PHLPP1 mRNA expression and 1.5 increase in PHLPP2. Impact of BMI‐1 on PHLPPs is not restricted to endometrial cancer HEC1A cells. The very similar results were obtained for breast cancer cells MDA‐MB‐231 (Figure S1). Down‐regulation of BMI‐1 in MDA‐MB‐231 cells caused decrease in pAKT level and increased PHLPP expression. In HEC1A cells, the BMI‐1 down‐regulation caused also decrease in *INPP5D* mRNA expression; however, the general expression of this gene in HEC1A cells is very low and this phosphatase does not seem to play a significant role in endometrial cells. The relative expression of genes coding for phosphatases are shown in Table S1.

### 3.2 Expression of BMI‐1, PTEN and pAKT in normal and cancer endometrial tissues

**Figure 2:**
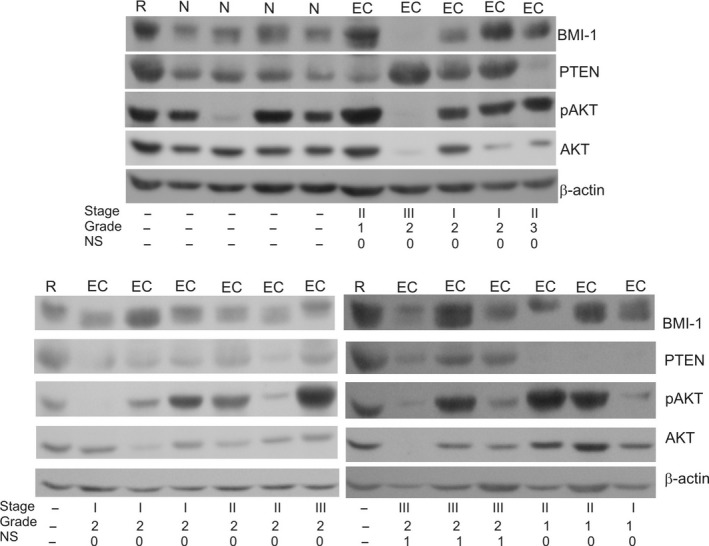
Representative results of Western blot analysis of BMI‐1, PTEN, AKT and phospho‐AKT levels in normal and cancer endometrial tissues. The stage, grade and lymph node metastasis status of cancers are indicated. R‐reference sample, N‐normal endometrial tissue, EC‐endometrial cancer, NS‐nodal status

Relative expression levels of BMI‐1, PTEN, AKT and level of AKT phosphorylation in 37 samples of normal endometrial tissues and 93 endometrial cancers were evaluated by Western blot and correlated with clinicopathological data. The representative results of Western blot analysis are shown in Figure 2. The results of densitometric analysis of all samples are shown in Table 2. There were no statistically significant differences between expression of BMI‐1 in non‐tumour tissues and low stage tumours (Table 2). However, in more advanced tumours, classified as stage III and IV, the expression of BMI‐1 was lower than in less advanced tumours, corresponding to the first and second stage according to FIGO classification (Table 2). The expression of BMI‐1 was also lower in cancers that exhibited the ability to metastasize to regional lymph nodes compared to non‐metastatic cancers (Table 2). There were no statistically significant differences of BMI‐1 protein expression between tumours of different histological grade. PTEN protein expression was found in all samples of normal tissue and 61 cancer samples (65.6%). Our results showed that PTEN protein expression was lower in PTEN positive tumour endometrial samples than in normal endometrial tissue samples (Table 2). However, there were no significant differences in PTEN protein expression between samples depending on FIGO stage or histological grade and lymph node metastasis status (Table 2).

**Table 2:** Associations between BMI‐1, PTEN, AKT and pAKT expressions and clinicopathological data

Clinicopathological parameter	BMI‐1		PTEN		AKT		pAKT		pAKT/AKT	
	Mean ± SD	*P‐*value	Mean ± SD	*P‐*value	Mean ± SD	*P*‐value	Mean ± SD	*P‐*value	Mean ± SD	*P‐*value
Normal	0.832 ± 0.115	.606	1.229 ± 0.356	**<.0001**	0.629 ± 0.062	0.051	1.795 ± 0.438	.093	3.059 ± 0.721	**.0029**
Cancer	0.752 ± 0.068		0.525 ± 0.105		0.701 ± 0.111		4.208 ± 0.974		17.43 ± 4.683	
Grade										
1	1.027 ± 0.190	.233	0.458 ± 0.092	.711	0.645 ± 0.164	0.661	4.639 ± 1.085	.142	5.775** ± **1.344	.860
2	0.714 ± 0.086		0.628 ± 0.182		0.736 ± 0.176		3.511 ± 0.727		13.91** ± **3.231	
3	0.665 ± 0.131		0.352 ± 0.071		0.674 ± 0.187		2.129 ± 0.688		10.08** ± **3.021	
Stage										
I	0.867 ± 0.104	**.0094** a	0.594 ± 0.175	.866	0.620 ± 0.105	0.386	5.465 ± 1.683	**.0015** b	13.34** ± **2.679	.073
II	0.826 ± 0.133		0.477 ± 0.188		0.781 ± 0.222		1.845 ± 0.466		7.457** ± **1.806	
III + IV	0.435 ± 0.072		0.408 ± 0.077		0.496 ± 0.134		0.771 ± 0.216		5.952** ± **2.112	
Lymph node metastasis										
No	0.826 ± 0.078	**.005**	0.552 ± 0.128	.710	0.582 ± 0.087	0.105	4.730 ± 1.158	**.021**	5.375 ± 1.086	**.015**
Yes	0.393 ± 0.083		0.394 ± 0.107		1.274 ± 0.478		1.695 ± 0.770		2.252 ± 0.252	

Note

Kruskal‐Wallis test was used to compare more than two groups. For pairwise multiple comparisons the Dunn's post hoc test was used.

Bold values show statistically significant differences.

a I vsIII + IV, II vs III + IV.

b I vsIII + IV, II vs III + IV.

There were no significant differences in expression level of AKT in samples of normal endometrial tissue and cancer samples or between cancers with different stage or grade (Table 2). Phosphorylation levels of AKT in endometrial tissue samples were expressed as pAKT to AKT ratio. In normal endometrial tissues, pAKT/AKT ratio was significantly lower than in the endometrial cancer tissues (Table 2). However, stage I tumours showed tendency to have higher pAKT/AKT ratio compared to higher stage tumours (*P* = .07). Lower phosphorylation was observed in cancers with ability to lymph node metastasis compared to those without metastasis (Table 2). No significant differences in pAKT/AKT ratio were observed between tumours of different histological grade.

### 3.3 Expression of *BMI1*, *PTEN*, *PHLPP1* and *PHLPP2* mRNA in normal and endometrial cancer samples

There were no statistically significant differences between *BMI1* mRNA expression in normal and cancer samples (Table 3). Less differentiated tumours showed a tendency to have higher BMI‐1 mRNA expression level than tumours of G1/G2. Similarly, to the protein expression results, there was a significantly lower BMI1 mRNA expression in cancers that exhibited the ability to metastasize to regional lymph nodes compared to non‐metastatic cancers.

**Table 3:** Associations between BMI1, PTEN, PHLPP1 and PHLPP2 mRNAs expression and clinicopathological data

Clinicopathological parameter	BMI‐1		PTEN		PHLPP1		PHLPP2	
	Mean ± SD	*P‐value*	Mean ± SD	*P‐value*	Mean ± SD	*P*‐value	Mean ± SD	*P*‐value
Normal	1760 ± 156	.08	8941 ± 1319	.051	7279 ± 1257	.135	885.0 ± 189.9	*.039*
Cancer	2504 ± 272		11 681 ± 2557		5803 ± 690.3		526.4 ± 76.33	
Grade								
1	1702 ± 179	.065	7011 ± 1865	*.013* a	5946 ± 1392	.807	540.3 ± 186.6	.509
2	3050 ± 106		12 514 ± 3106		6198 ± 1005		538.6 ± 106.3	
3	3920 ± 350		3465 ± 843.3		4520 ± 951.3		478.5 ± 118.5	
Stage								
I	3076 ± 450	.09	7632 ± 1124	*.012* b	6951 ± 1002	.091	602.4 ± 108.9	.284
II	2750 ± 206		2820 ± 645.5		3873 ± 851.7		317.8 ± 62.4	
III + IV	2078 ± 388		5458 ± 1523		4784 ± 1592		557.2 ± 213.4	
Lymph node metastasis								
No	2432 ± 276	**.046**	11 400 ± 2775	.905	5862 ± 738.0	.425	507.2 ± 77.7	.950
Yes	1690 ± 305		6554 ± 1949		5474 ± 2003		633.1 ± 262.2	

Note

Kruskal‐Wallis test was used to compare more than two groups. For pairwise multiple comparisons the Dunn's post hoc test was used.

Bold and italic values show statistically significant differences.

a 2 vs 3.

b I vs II.

As BMI‐1 protein is suggested to be a negative regulator of PTEN gene transcription, we analysed additionally to PTEN protein also PTEN mRNA expression level. Changes in gene transcription may be poorly reflected at the protein level because protein level depends also on post‐translational modifications and protein half‐life. Indeed, the mRNA and protein level of PTEN showed only moderate correlation in endometrial tissues (Spearman *r* = .476, *P* = .0022). Our results showed tendency of higher *PTEN* mRNA expression in tumour endometrial samples compared to normal endometrial tissue samples (*P* = .051) (Table 3). Expression levels of PHLPP1 and PHLPP2 mRNAs in samples of endometrial cancers and normal endometrial tissues were also analysed by RT‐PCR and correlated with clinical and pathomorphological data (Table 3). Analysis showed that the mean mRNA expression of PHLPP2 was lower in cancer samples than in normal tissue. However, there were no significant differences in PHLPP1 or PHLPP2 expression between samples depending on FIGO stage or histological grade and lymph node metastasis status (Table 3).

### 3.4 Correlation between BMI‐1, PTEN, PHLPP1/2 expression and AKT phosphorylation level

The correlations between BMI‐1, PTEN, PHLPPs and phosphorylation level of AKT were analysed in order to check potential association of BMI‐1 with PTEN/AKT pathway. We did not observe correlation between BMI‐1 and PTEN proteins expression levels (Table 4). Significant positive correlation was observed between expression of BMI‐1 and AKT phosphorylation level (pAKT/AKT) in normal and cancer endometrial samples (0.598 *P* = .0002, .559, *P* = .0001). Significant strong inverse correlation was found between BMI‐1 and PHLPP1/2 in normal samples. Inverse correlation was found in cancer samples between pAKT/AKT and PTEN, but the correlation was shown only in less advanced cancers stage I and non‐metastatic cancers (Table 4).

**Table 4:** Correlation between BMI‐1, PTEN, pAKT and PHLPP1/2 expressions

	BMI‐1/PTEN		BMI‐1/pAKT		PTEN/pAKT		BMI‐1/PHLPP1		BMI‐1/PHLPP2	
	Spearman *r*	*P*‐value	Spearman *r*	*P*‐value	Spearman *r*	*P*‐value	Spearman *r*	*P*‐value	Spearman *r*	*P*‐value
Normal	.129	.473	**.598**	**.0002**	−.018	.919	−**.622**	**<.001**	−**.556**	**<.001**
Cancer	−.165	.152	**.559**	**<.0001**	−**.370**	**.001**	−.098	.413	−.089	.452
Cancer PTEN+	−.237	.117	**.580**	**<.0001**	−.291	.052	−**.396**	**.003**	−**.339**	**.007**
Cancer PTEN−	‐	**‐**	**.440**	**.0021**	‐	‐	−.146	.442	−.034	.856
Stage I	−.092	.559	**.623**	**<.0001**	−**.403**	**.008**	−.230	.146	−.155	.337
Stage II‐IV	−.255	.145	**.437**	**.0097**	−.296	.089	.002	.991	−.038	.837
Nodal metastasis −	−.173	.176	**.506**	**<.0001**	−**.384**	**.002**	−.206	.114	−.137	.294
Nodal metastasis +	−.200	.493	**.665**	**.0093**	−.481	.081	.273	.391	.111	.729

Bold values show statistically significant differences.

Since relationship between PTEN and PHLPP expression was suggested in some cells,^14^ we analysed also correlation between BMI‐1 and PHLPPs separately in PTEN positive and negative cancers. PTEN protein expression was found in all samples of normal tissue and 61 cancer samples (65.6%). There was no correlation between BMI‐1 and PHLPP1 or PHLPP2 in PTEN negative cancers (*P* > .05), but weak/moderately strong inverse correlation was observed in case of PTEN positive cancers. Moderately strong inverse correlation was observed between PTEN and PHLPPs only in case of cancer samples (Table 4).

### 3.5 PTEN, BMI‐1 and PHLPPs status and endometrial cancer prognosis

**Figure 3:**
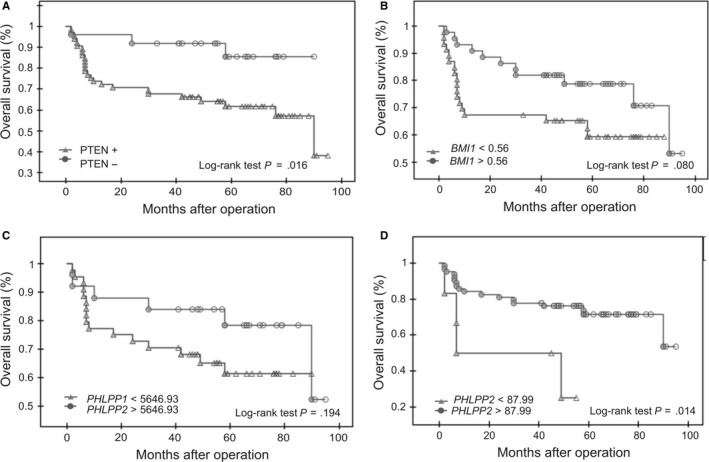
PTEN, BMI‐1 and PHLPP1/2 expression status and endometrial cancer prognosis. Kaplan‐Meier curve showing overall survival for endometrial cancer patients with positive and negative expression of PTEN (A); low and high levels of BMI‐1 protein expression (B); low and high PHLPP1 or PHLPP2 mRNA expression (C and D, respectively). Significance was estimated with the log‐rank test. Overall survival was defined as the interval from the date of initial surgical resection to the date of last known contact of death

Kaplan‐Meier survival analysis was used to estimate the predictive effect of PTEN, BMI‐1 and PHLPPs expressions on overall survival (Figure 3). Overall survival differences in case of BMI‐1 and PHLPP1 were not statistically significant. However, there was a tendency that patients in the low BMI‐1 expression group had a shorter overall survival than those in the high BMI‐1 group (log‐rank test *P* = .08, Figure 3B). The Kaplan‐Meier analysis showed that patients in the low PHLPP2 expression group had a significantly shorter overall survival than those in the high PHLPP2 group (log‐rank test, *P* = .014, Figure 3D). Moreover, patients in the positive PTEN expression group had a significantly shorter overall survival than those in the negative PTEN expression group (log‐rank test *P* = .016, Figure 3A).

## 4 DISCUSSION

Increased activity of PI3K/AKT pathway found in many human cancers can be the result of overexcitation at the receptor level, loss of inhibiting function of *PTEN* and amplification or mutation in *PI3K* or *AKT* genes. Alteration of this pathway is one of the best‐recognized abnormalities in endometrial cancer.^10^ It is suggested that low PTEN protein expression may be involved in pathogenesis and development of endometrial carcinoma.^15^ PTEN protein level can be altered upstream at the genetic level through mutation, but also at the transcriptional level or post‐translational level. Recent studies suggest that in some cancer cell lines polycomb‐group protein BMI‐1 negatively regulates PTEN expression and promotes cancer progression due to increased activity of AKT.^3^, ^5^, ^16^ BMI‐1 depletion was shown to cause EMT inhibition in nasopharyngeal and melanoma cells.^3^, ^17^ BMI‐1 promoted also invasion and metastasis of hepatocellular carcinoma and pancreatic cancer stem cells.^4^, ^5^ However, in contrast to that Xiong et al^18^ indicated that low BMI‐1 expression might contribute to the metastasis of lung cancer. They found that silencing of BMI‐1 in A549 cells and H292 cells led to decreased expression of epithelial markers and concomitant enhanced expression of mesenchymal markers. Thus, function of BMI‐1 in cancer may be cell‐context dependent.

The objective of our study was to determine the expression of BMI‐1 protein in endometrial cancer and determine whether its expression inversely correlates with PTEN gene expression and positively correlate with AKT phosphorylation level. BMI‐1 was observed to be up‐regulated in a variety of tumours, but data on the expression of BMI‐1 protein in endometrial cancer are very limited and inconsistent. Honig et al^6^ found that endometrial carcinomas express higher level of BMI‐1 than benign controls. However, they compared relatively low number of samples (12 samples of endometrial carcinoma and 6 samples of benign endometrial tissues). Engelsen et al^9^ studied BMI‐1 expression by immunohistochemistry in a large group of samples from endometrial cancer patients, but benign endometrial tissue samples were not compared to malignant tissue in this study. They demonstrated that low BMI‐1 expression was associated with an aggressive phenotype in endometrial carcinomas.

The results of our studies did not show significant difference between the low‐stage endometrial cancers and normal tissue. However, we found lower expression of BMI‐1 in samples of more advanced endometrial carcinomas (stage III/IV) compared to less advanced tumours (stage I and II). Moreover, the expression of BMI‐1 was lower in tumours that exhibited the ability to metastasize to regional lymph nodes compared to non‐metastatic cancers. Thus, our results similarly to results of Engelsen et al^9^ suggest that low expression of BMI‐1 correlates with a more aggressive phenotype of endometrial carcinoma. We used Kaplan‐Meier survival analysis to estimate the predictive effect of BMI‐1 expression on overall survival. There was a tendency that patients in the low BMI‐1 expression group had a shorter overall survival than those in the high BMI‐1 group.

In many studies, PTEN protein level in endometrial cancers have been analysed by immunohistochemistry, but the results regarding PTEN expression impact on cancer progression and prognosis are inconsistent. Some studies showed that lack or low PTEN level is associated with worse prognosis,^19^, ^20^, ^21^ but the others suggest that loss of PTEN expression is associated with favourable prognosis.^22^, ^23^, ^24^ Gao et al^24^ found that the level of PTEN expression in patients with endometrial carcinoma was significantly related to differentiation (the expression of PTEN in high differentiated endometrial carcinoma was significantly higher than that in middle‐low differentiated one and clinical stage). In our study, PTEN protein expression was much lower in tumours compared to controls, but we did not find significant differences between tumours of different clinical stage or grade.

Our results did not show correlation between BMI‐1 and PTEN mRNA or protein levels which together with results of in vitro study seemed to exclude the possibility that BMI‐1 regulates PTEN expression in endometrial cells. However, we found positive correlation between BMI‐1 expression and AKT phosphorylation level. Similarly to BMI‐1 lower expression of pAKT in more advanced cancers (FIGO stage III/IV) than less advanced ones was found. Low levels of AKT phosphorylation was found also in cancers with ability to metastasize. Given the function of AKT and BMI‐1 as oncogenesone would have expected that decreased AKT phosphorylation would be associated with inhibition of metastasis. But there are several studies suggesting that AKT activation can promote tumorigenesis but suppresses tumour invasion.^25^, ^26^, ^27^, ^28^ Thus, paradoxically low pAKT may favour metastasis.

BMI‐1 as a negative transcription regulator may suppress activity of the other proteins involved in AKT regulation. Since phosphorylation of AKT may be affected directly or indirectly by several phosphatases, that is PHLPP, PP2A, SHIP,^28^, ^29^, ^30^ we analysed the effect of BMI‐1 depletion in HEC1A cells on their expression. The results showed increased expression of PHLPP, especially PHLPP1 isoform. Also, we found the strong inverse correlation between BMI‐1 and PHLPP1/2 in normal endometrial tissues. Weak inverse correlation has been found only in cancers with expression of PTEN. These results may suggest for the first time that BMI‐1 impact on PHLPP and at least partially it is dependent on PTEN.

PHLPP1 and PHLPP2 have been identified as tumour suppressors because they can suppress of pro‐survival signalling, such as PI3K/Akt signalling pathway. According to that both PHLPP1 and PHLPP2 expressions are lost in diverse cancers,^31^ but their role in progression of cancers has not been identified. Our results are the first results concerning PHLPP1 and 2 expressions in endometrial cancer. We found decreased expression of PHLPP2 in cancer samples compared to non‐tumoral samples, but there were no significant differences in PHLPP expression between samples depending on FIGO stage or histological grade and lymph node metastasis status. However, PHLPP2 seems to be a promising predictive factor of patients overall survival (Figure 3, log‐rank test *P* = .014). Thus, further analysis of PHLPPs expression especially on protein level including higher number of cases should be performed. Unfortunately, the commercially available antibodies against PHLPP should be used with caution since it is suggested that some PHLPP1 antibodies recognize a signal in tissues that may represent a non‐specific band unrelated to PHLPP1, that cover the specific one.^32^

In summary, BMI‐1 affects AKT activity by regulation of PHLPP expression in endometrial cells. Impact of BMI‐1 on PHLPP in endometrial cancer probably depends on PTEN since correlation between BMI‐1 and AKT phosphorylation exist only in PTEN positive normal and cancer tissues. Understanding the relationship between PI3K/AKT pathway and BMI‐1 function may contribute in the future to the development of therapeutic approaches that will be better adapt to the molecular context and the specificity of endometrial cancers. Our analyses shed new insights into molecular mechanisms of BMI‐1, PHLPP and pAKT interdependence underlying endometrial cancer progression, and they offer implications for prognosis and drug selection strategies. Our current findings suggest that low BMI‐1 expression in endometrial cancer can be a potential biomarker of aggressive tumour and poor prognosis in endometrial cancer patients. Moreover, we suggest that assessment of combined expression of BMI‐1, PTEN and PHLPP may be especially helpful in endometrial cancer prognosis. Despite the fact that overexpression of BMI‐1 is involved in promotion of invasion and metastasis of several cancers, its role in endometrial cancer seems to be different. These findings suggest that drugs based on negative regulation of BMI‐1 protein expression such as PTC‐596 which is currently in clinical development and is used in patients with advanced solid tumours (NCT02404480), may be not suitable for advanced endometrial cancer.

In summary, our results for the first time showed that down‐regulation of BMI‐1 in cancer cells might affect expression of PHLPP1 and PHLPP2. Moreover, decreased PTEN and increased phospho‐AKT levels are more likely associated with an early event in endometrial tumorigenesis, while low expression of BMI‐1 and low level of AKT phosphorylation may be involved in endometrial cancer progression. BMI‐1 seems to impact on AKT phosphorylation level in endometrial cells by regulation of PHLPP expression. However, some limitations of our work need to be considered. First one is the small sample size, especially the small number of more advanced and aggressive cancer samples. Further studies are needed for larger group of patients with stage III and IV cancer. The second limitation is that analyses of association between studied genes and proteins were performed using only one endometrial cancer cell line. Although, we obtained the similar results for breast cancer cells, to verify the universality of the mechanism in endometrial cancer and thus its significance, other endometrial cancer cell lines should be investigated. Taking into account that relationship between BMI‐1 and PHLPP seems to be dependent on PTEN expression it is necessary to use in future studies both PTEN positive and PTEN negative cells. Third, we have not taken into account mutations in studied genes, especially those, that do not affect expression but affect the activity of proteins. It should be done in future to find out whether relationship between BMI‐1 and PHLPP depends on PTEN enzymatic activity.

## CONFLICT OF INTEREST

The authors confirm that there is no conflict of interest.

## AUTHOR CONTRIBUTIONS

AZ, AK and PJ involved in study conception and design. ŁC, KWK and PC involved in acquisition of data. AZ, PC, EF and PJ involved in analysis and interpretation of data. AK and AZ drafted the manuscript. MB and AB critically revised the manuscript.

## Data Availability

The data that support the findings of this study are available from the corresponding author upon reasonable request.
