# Supplementary material for: Relationship between polycomb‐group protein BMI‐1 and phosphatases regulating AKT phosphorylation level in endometrial cancer
Source: J Cell Mol Med. 2019 Dec 21;24(2):1300–10. doi: 10.1111/jcmm.14782 (PMC6991679; doi:10.1111/jcmm.14782)
Supplement: Supplementary file 1 [file JCMM-24-1300-s001.docx]

**Relationship between polycomb group protein BMI-1 and phosphatases regulating AKT phosphorylation level in endometrial cancer**

Agnieszka Zaczek^1^, Paweł Jóźwiak^1^, Piotr Ciesielski^1^ , Ewa Forma^1^, Katarzyna Wójcik-Krowiranda^2^, Łukasz Cwonda^2^, Andrzej Bieńkiewicz^2^, Magdalena Bryś^1^, Anna Krześlak^1^

^1^Department of Cytobiochemistry, Faculty of Biology and Environmental Protection,

University of Lodz, Pomorska 141/143, 90-236 Lodz, Poland

^2^Clinical Division of Gynecological Oncology, Medical University of Lodz, Pabianicka 62, 93-513 Lodz, Poland

Corresponding author: Anna Krześlak, University of Lodz, Faculty of Biology and Environmental Protection, Department of Cytobiochemistry, Pomorska 141/143, 90-236 Lodz

e-mail: krzeslak@biol.uni.lodz.pl

Tel. +48426354371

Fax. +48426354484

**Supplemantary Figure 1.** Effect of BMI-1 downregulation on AKT phosphorylation level and expression of phosphatases in MDA-MB-231 cells. A) Representative immunoblots showing BMI-1, PTEN, AKT proteins and phosphorylated AKT in MDA-MB-231 cells treated for 48h with 30 nM BMI-1 siRNA or scrambled siRNA (control). B) Bar graph shows the densitometric analysis of BMI-1, PTEN, AKT and phosphorylated AKT (pAKT) levels in cells treated with BMI-1 siRNA and scrambled siRNA and represents the mean ± SD of three independent experiments. C) Relative changes in BMI1, PP2A, PHLPP1,PHLPP2, INPP4B, INPP5D and PTEN mRNAs expression levels in siRNA treated cells compared to untreated cells; bar graph represents the mean ±SD.*p< 0.05, **p<0.01

**Supplemantary Table 1.** mRNA expression of genes coding for phosphatases in HEC1A and MDA-MB-231 cells. The relative expression levels of genes were calculated using ΔCt method. ΔCt (Ctgene—CtHPRT) values were recalculated into relative copy number values (number of gene mRNA copies per 1000 copies of *HPRT1* mRNA). Relative genes expression in cells was calculated using ΔΔCT normalized to HPRT1 gene expression.

| **Phosphatases** | **HEC-1A**  Mean ±SD p*-value* | **MDA-MB-231**  Mean ±SD *p-value* |
| --- | --- | --- |
| ***BMI1***  *Control*  *siBMI1*  ***PP2A***  *Control*  *siBMI1*  ***PHLPP1***  *Control*  *siBMI1*  ***PHLPP2***  *Control*  *siBMI1*  ***INPP4B***  *Control*  *siBMI1*  ***INPP5D***  *Control*  *siBMI1*  ***PTEN***  *Control*  *siBMI1* | 386.0±9.0 0.0023  97.62±10.5  5909±368.1 0.9241  5840±524.8    126,4±5.7 0.0080  249.3±9.**5**    386.1±13.96 0.0116  588.9±22.44  113.0±27.63 0.4674  88.40±1.225  0.620±0.06 0.0203  0.200±0.010  1723±29.86 0.073  2227±7.718 | 807.9±32.85 0.0001  248.8±19.18  2690±273.5 0.2713  3114±188.5  55.93±9.2 0.019  144.4±3.9    293.2±19.7 0.0234  389.6±18.8  790.6±42.56 0.0247  1076±69.41  0.2433±0.02 0.2741  0.313±0.05  3828±628.2 0.3374  4759±131.9 |

**Supplenmentary Figure 2.** Full blots to Figure 2.

**Blots to Figure 2 (TOP)**


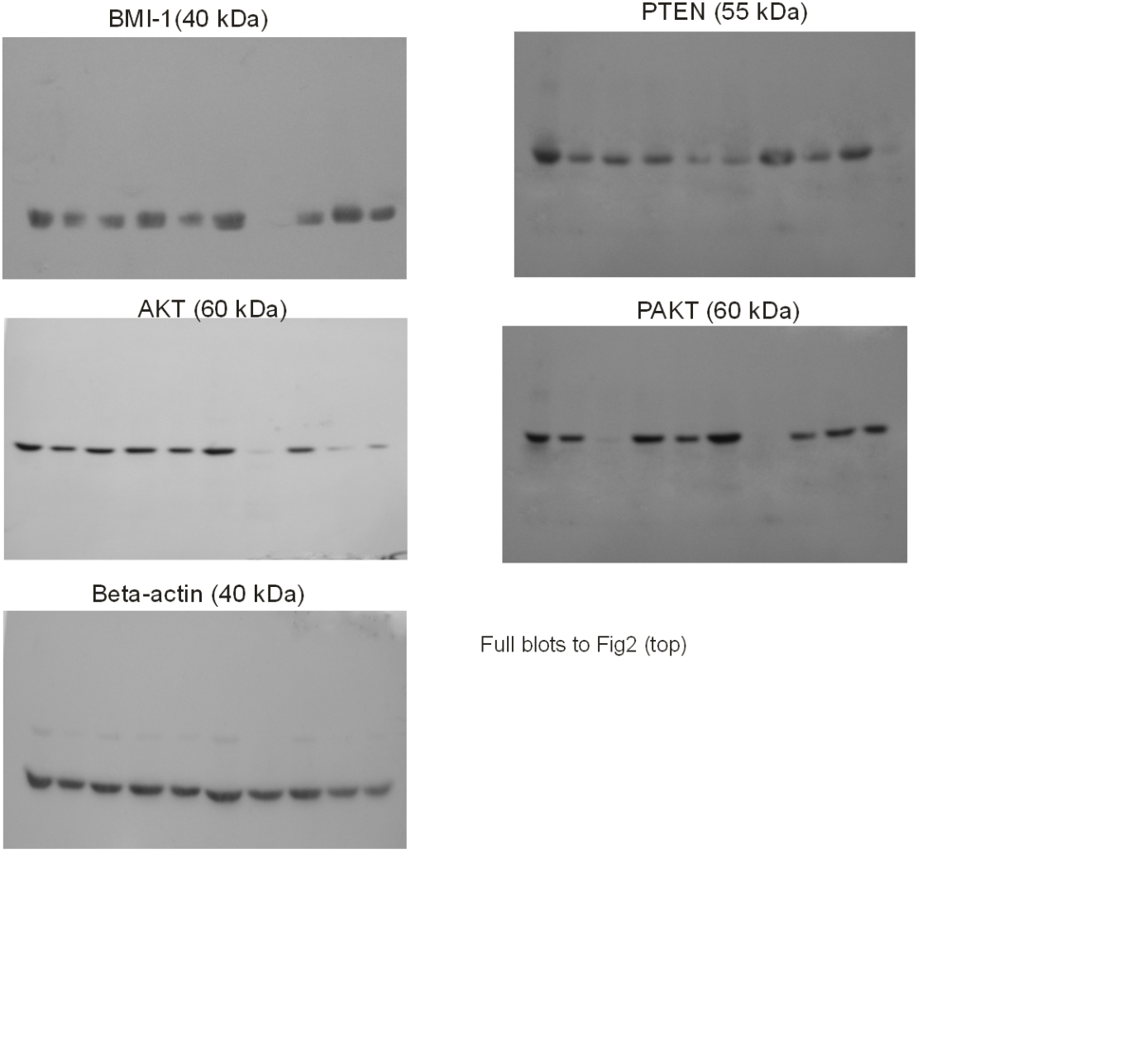


**Blots to Figure 2 (LEFT)**

**
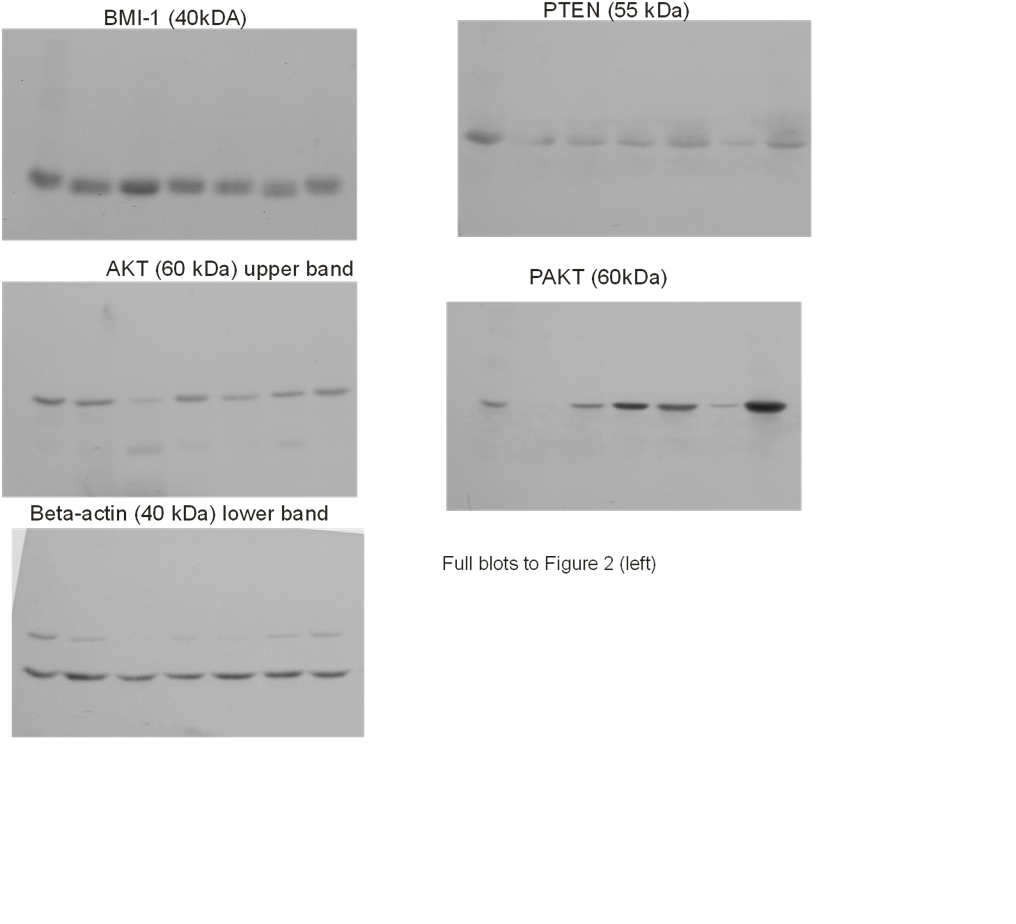
**

**Blots to Figure 2 (RIGHT)**

**
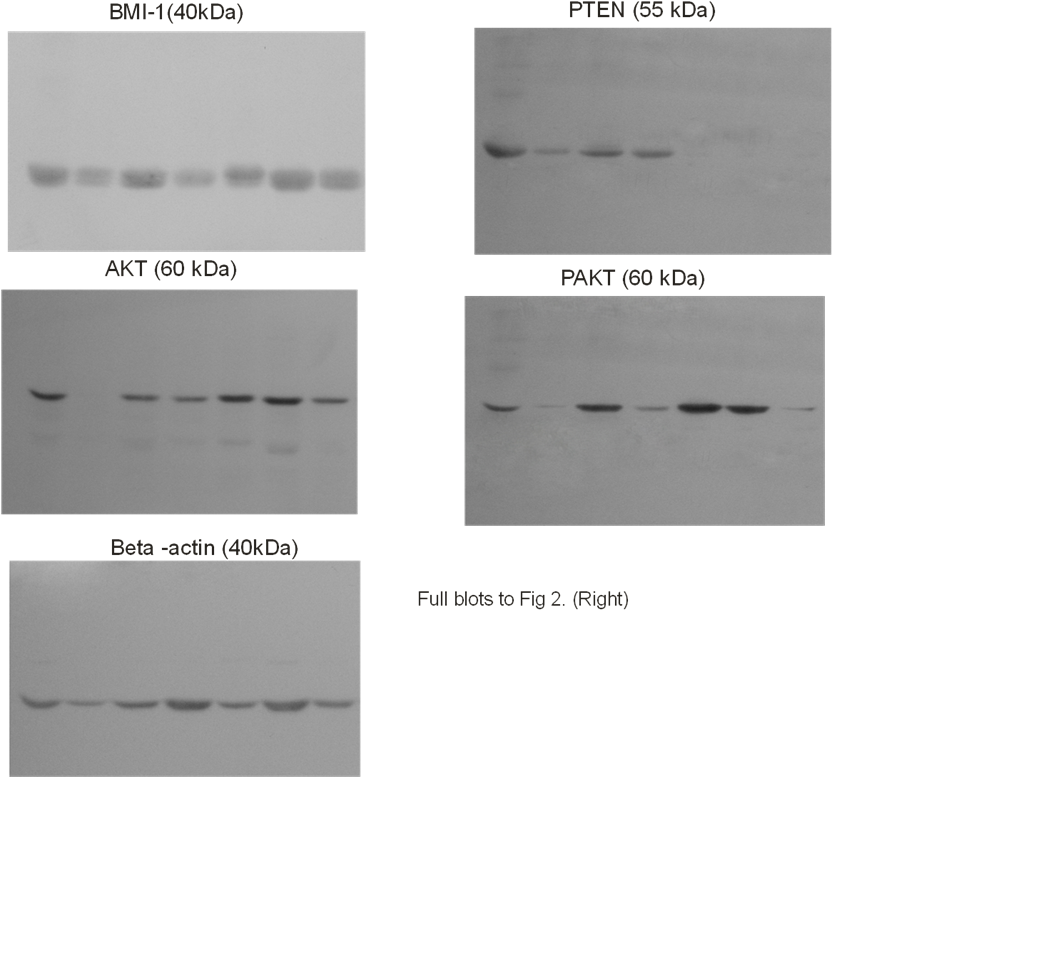
**
